# Commentary Unveiling the prognostic potential of C-reactive protein-to-albumin ratio in valvular heart disease: a step toward personalized risk stratification

**DOI:** 10.1097/JS9.0000000000003542

**Published:** 2025-10-09

**Authors:** Yuru Fu, Yuanyuan Wang

**Affiliations:** a Department of Intensive Care Unit, Fuyang People’s Hospital of Anhui Medical University, Fuyang, China; b Department of Cardiology, Fuyang People’s Hospital of Anhui Medical University, Fuyang, China

*Dear Editor,*

Valvular heart disease (VHD) remains a significant contributor to cardiovascular morbidity and mortality worldwide, particularly as populations age and comorbidities accumulate. The quest for reliable, accessible biomarkers to enhance risk stratification and guide therapeutic decisions has been ongoing. This commentary adheres to the TITAN Guidelines 2025 for the declaration and use of artificial intelligence (AI) in scientific publications, and no AI tools were used in the design, analysis, or writing of this work^[1]^. In this context, the recent nationwide prospective cohort study by Zhang *et al*^[2]^ offers compelling insights into the prognostic value of the C-reactive protein (CRP)-to-albumin ratio (CAR) in patients with moderate or severe VHD. Drawing from a large cohort of 5519 patients across various VHD subtypes – including aortic stenosis (AS), aortic regurgitation (AR), mitral stenosis (MS), mitral regurgitation (MR), tricuspid regurgitation (TR), and multivalvular heart disease (MVHD) – the study demonstrates that elevated CAR levels are independently associated with increased all-cause mortality and major adverse cardiovascular events at 2-year follow-up.

The core finding of Zhang *et al*^[2]^ is the identification of CAR as a composite biomarker that integrates inflammatory and nutritional status, outperforming CRP or albumin alone in prognostic utility. Using penalized splines and receiver operating characteristic curves, the authors established an optimal CAR threshold of 0.15, above which patients exhibited a monotonic increase in relative hazards for mortality (adjusted hazard ratio [aHR]: 1.89; 95% CI: 1.56–2.28; *P* < 0.001). This association held across most VHD subtypes, with notable excess mortality in AS (aHR: 3.30; 95% CI: 1.83–5.96), AR (aHR: 1.98; 95% CI: 1.18–3.32), MR (aHR: 1.33; 95% CI: 1.03-–1.71), TR (aHR: 1.82; 95% CI: 1.35–2.46), and MVHD (aHR: 1.34; 95% CI: 1.13–1.60), though it was not significant in MS, possibly due to the low event rate (only seven deaths) and the younger demographic often receiving early interventions. Intriguingly, combining CAR with N-terminal pro-B-type natriuretic peptide (NT-proBNP) amplified risk stratification, with patients exhibiting elevations in both markers facing a 23.5% cumulative 2-year mortality rate – far exceeding those with isolated elevations (10.7% for high NT-proBNP alone and 5.1% for high CAR alone). The addition of CAR to traditional risk models improved prediction metrics, yielding a continuous net reclassification index of 0.57 (*P* < 0.001) and an integrated discrimination improvement of 0.014 (*P* < 0.001), underscoring its incremental value.

This study builds on prior evidence linking inflammation and malnutrition to cardiovascular outcomes. For instance, CRP has long been recognized as a predictor in conditions like atherosclerotic disease and heart failure^[3,4]^, while hypoalbuminemia correlates with adverse events post-transcatheter aortic valve replacement^[5]^. By merging these into CAR, Zhang *et al*^[2]^ address a gap in VHD prognostication, where single biomarkers often fall short due to the multifactorial nature of the disease. The cohort’s diversity – encompassing rheumatic, degenerative, congenital, ischemic, and functional etiologies – enhances generalizability within the Chinese population, and the use of inverse probability treatment weighting to adjust for confounders like age, comorbidities, and valvular interventions strengthens the robustness of the findings. Sensitivity analyses excluding patients with low body mass index (≤18 kg/m^2^) or chronic diseases further affirm CAR’s independent prognostic role, mitigating concerns about confounding from systemic inflammation or malnutrition unrelated to VHD.

Despite these strengths, several limitations warrant consideration, as acknowledged by the authors. The exclusion of over half the original registry cohort due to missing CRP or albumin data introduces potential selection bias, with included patients showing slightly higher cardiometabolic comorbidities – possibly reflecting clinician-driven testing in higher-risk individuals. Baseline-only CAR measurements preclude assessment of dynamic changes, which could refine timing for interventions like surgery. Echocardiographic data were site-reported rather than core-lab adjudicated, risking variability, though quality control measures were implemented. The focus on Chinese patients limits ethnic generalizability, and unaccounted medication effects (e.g., anti-inflammatory drugs) could influence CRP/albumin levels. Moreover, while CAR’s prognostic value is clear, its role in guiding surgical timing remains exploratory; extreme elevations might contraindicate immediate intervention due to heightened perioperative risks^[6]^.

Looking ahead, this work paves the way for biomarker-driven personalization in VHD management. Future studies should explore serial CAR monitoring to optimize surgical windows, validate the 0.15 threshold in diverse global cohorts, and integrate CAR into multimarker panels alongside imaging modalities^[7]^. Randomized trials assessing CAR-guided strategies could determine if they reduce mortality compared to standard care. In an era of precision medicine, CAR emerges as a simple, cost-effective tool to navigate the “intricate maze” of VHD prognostication, potentially improving outcomes by identifying at-risk patients earlier.

In summary, Zhang *et al*^[2]^ provide a timely advancement in VHD risk assessment, highlighting CAR’s potential to enhance stratification and decision-making. While challenges remain, this biomarker holds promise for routine clinical integration, bridging inflammation, nutrition, and prognosis in a field ripe for innovation.

## Data Availability

No other datasets were generated during and/or analyzed during the current study. All the information is available with the manuscript.
